# Advance of Clustered Regularly Interspaced Short Palindromic Repeats-Cas9 System and Its Application in Crop Improvement

**DOI:** 10.3389/fpls.2022.839001

**Published:** 2022-05-12

**Authors:** Yuchun Rao, Xi Yang, Chenyang Pan, Chun Wang, Kejian Wang

**Affiliations:** ^1^College of Chemistry and Life Sciences, Zhejiang Normal University, Jinhua, China; ^2^State Key Laboratory of Rice Biology, China National Rice Research Institute, Hangzhou, China

**Keywords:** CRISPR-Cas9 system, gene knockout, application, crop improvement, future development

## Abstract

Clustered regularly interspaced short palindromic repeats (CRISPR)-Cas9 is the third generation of novel targeted genome editing technology after zinc finger nucleases (ZFNs) and transcription activator like effector nucleases (TALENs). It is also one of the most promising techniques for mutating and modifying genes. The CRISPR-Cas9 system has the advantages of simplicity, high efficiency, high specificity, and low production cost, thus greatly promoting the study of gene function. Meanwhile, it has attracted the attention of biologists. After the development and improvement in recent years, CRISPR-Cas9 system has become increasingly mature and has been widely used in crop improvement. Firstly, this review systematically summarizes the generation and advantages of CRISPR-Cas9 system. Secondly, three derivative technologies of the CRISPR-Cas9 system are introduced. Thirdly, this review focuses on the application of CRISPR-Cas9 system in gene knockout, gene knock-in, and gene regulation, as well as the improvement of yield, quality, and biological resistance of important crops such as rice, wheat, soybean, corn, and potato. Finally, this review proposes the potential challenges of CRISPR-Cas9 system, and discusses the future development of CRISPR-Cas9 system.

## The Production and Advantages of CRISPR-Cas9 Technology

Ishino et al. (1987) first discovered the existence of special repeat sequences separated by spacer sequences of similar size in the *Escherichia coli* genome when he was studying the *E. coli*. Jansen et al. (2002) named it as the Clustered Regularly Interspaced Short Palindromic Repeats (CRISPR), and at the same time found CRISPR-associated (*Cas*) gene, near the CRISPR sequence, and analyzed its function. Barrangou et al. (2007) discovered an acquired immunity against phage and other exogenous genes mediated by CRISPR sequence and Cas protein in prokaryotes. Jinek et al. (2012) elucidated the mechanism of action of CRISPR-Cas. Based on the acquired immune system, Church’s laboratory modified the system to construct a type II CRISPR/Cas system, namely the CRISPR-Cas9 system, which has become a technology that can edit DNA in animal and plant cells (Mali et al., 2013). The two main elements of the CRISPR-Cas9 system are guide RNA (sgRNA) and Cas9 protein. The target genes of the CRISPR-Cas9 system usually contain a short Protospacer adjacent motif (PAM), and the PAM sequence is usually NRG (R = G/A). SgRNA is an RNA sequence designed according to the target gene and has the function of guiding the Cas9 protein to cut the target gene. Cas9 protein is an endonuclease containing RuvC and HNH domains, which are responsible for cleaving the target strand and non-target strand of the target gene, respectively. The cleavage site is located 3 ~ 8 nt upstream of PAM (Friedland et al., 2013). The working mechanism of the CRISPR-Cas9 system is that the Cas9 protein is guided by sgRNA to introduce a DNA double-strand break (DSB) into a specific target gene. Once this break occurs in the cell, it will trigger homologous recombination (HR) or non-homologous end joining (NHEJ) two automatic repair mechanisms, resulting in the insertion, deletion, and replacement of target gene sequences, to achieve directional modification of the genome (Jinek et al., 2012; Li et al., 2013). Genome editing technology is a technology for studying gene function and targeted modification of the genome. CRISPR-Cas is the third generation of new targeted genome editing technology after zinc finger nucleases (ZFNs) and transcription activator like effector nucleases (TALENs). CRISPR-Cas9 technology is the most widely used type of CRISPR-Cas system. Both ZFNs and TALENs are composed of a specific DNA binding domain and a non-specific cleavage domain (endonuclease FokI). CRISPR-Cas9 is also composed of a specific sgRNA and a non-specific endonuclease Cas9. In terms of specificity and off-target effects, CRISPR-Cas9 is stronger than ZFN, but slightly worse than TALEN. In terms of design and construction system, CRISPR-Cas9 is simpler in design than ZFN and TALEN. In addition, CRISPR-Cas9 has low production cost, not restricted by methylation, and can edit multiple genes at the same time (Li et al., 2013; Kumar et al., 2016; Liu et al., 2016). All things considered, CRISPR-Cas9 is the first choice when using sequence-specific nucleases for gene editing.

## Derivative Technology of CRISPR-Cas9 Technology

### CRISPR-dCas9 System

The CRISPR-dCas9 system was born in 2013. Qi et al. (2013) mutated the two conserved endonuclease domains of the Cas9 protein in the CRISPR-Cas9 system, namely the RuvC domain and the HNH domain. Cas9 protein loses endonuclease activity and cannot cut DNA, but still retains the ability to bind to DNA sequences. The CRISPR-dCas9 system usually plays a role in the process of gene regulation. The properties of dCas that can bind to double-stranded target genes are similar to transcription factors that regulate gene expression, but dCas cannot function alone. The purpose of activating or inhibiting gene transcription can be achieved by combining dCas with transcriptional activator protein/transcriptional repressor protein. In addition, although dCas cannot function alone, it can cause steric hindrance and prevent gene transcription.

### Base Editor

Base editors (BEs) include single base editors and double base editors.

Single base editors include cytidine base editors (CBEs) and adenine base editors (ABEs). The basic components of the CBE system include: sgRNA, nicking Cas9 (nCas9) protein with single-strand cleavage activity, cytosine deaminase, and uracil glycosylase inhibitor (UGI). The specific working mechanism is: nCas9 cleaves the non-target strand under the guidance of sgRNA, and cytosine (C) in the editing window is catalyzed by cytosine deaminase to remove an amino group and mutate to uracil (U), and UGI prevent the base U from being excised. During DNA replication, U is recognized as thymine (T), which is complementary to adenine (A), and in the next round of DNA replication, A and T normal pairing to achieve transformation of C to T (Komor et al., 2016). The ABE system is composed of sgRNA, nCas9 protein with single-strand cleavage activity and adenine deaminase fused. The working principle is: nCas9 cuts the non-target strand under the guidance of sgRNA, and adenine deaminase catalyzes the removal of an amino group from A in the target gene editing window to become inosine (I). It is recognized as guanine (G) during DNA replication, which is complementary to C. In the next round of DNA replication, G is paired with C, which finally completes the A to G transition (Gaudelli et al., 2017).

Double base editor is the Saturated Targeted Endogenous Mutagenesis Editor (STEME); this double base editor can induce C to T and A to G mutations at the target site at the same time under the guidance of a sgRNA, significantly increasing the saturation and diversity of base mutations (Li et al., 2020a).

### Prime Editor

The prime editor (PE) was developed in 2019 and theoretically enables 12 substitutions of four types of bases. The main elements include prime editing extended guide RNA (pegRNA), nSpCas9 (H840A), and moloney murine leukemia virus (M-MLV) reverse transcriptase. Among them, pegRNA is a modification of gRNA, adding a primer-binding sequence (PBS) and a reverse transcription template (RT) to its 3′ end, and adding editing information to the reverse transcription template, and nSpCas9 is a modification of Cas9 so that it only has cleavage single-stranded DNA activity. The PE is that the gRNA is paired with the target gene, instructing nSpCas9 to cut the target strand, generating a single-strand break nick, PBS binds to the 3′ end of the break nick, bringing RT to the nick. And then, under the action of MMLV, using RT as a template, a single-stranded DNA sequence is synthesized from the 3′ end of the broken nick. The automatic repair mechanism of the organism is triggered, and the newly synthesized DNA sequence is used as a template to repair the other DNA chain, thereby in any base substitution is introduced on the double strand of DNA (Anzalone et al., 2019).

## CRISPR-Cas9 Application

In recent years, the research on the CRISPR-Cas9 system has been applied in various forms, including applications in gene knockout, gene knock-in, and gene regulation. Among them, gene knockout is the most widely used (Zhao et al., 2019). Table 1 summarizes the applications of CRISPR-Cas9 in these three aspects.

**Table 1:** Clustered regularly interspaced short palindromic repeats (CRISPR)-Cas9 application.

Species	Target gene(s)	Gene function	CRISPR-Cas9 technical principle	Mutation mode	Mutant features	References
Single gene one site knockout						
Tomato	*SlLBD40*	Lateral organ boundaries domain transcription factor	CRISPR-Cas9 knockout vector	Gene knockout	Strong drought resistance	Liu et al., 2020b
Maize	*Zmzb7*	Encodes an IspH protein that plays an important role in the methyl-D-erythritol-4-phosphate (MEP) pathway	CRISPR-Cas9 knockout vector	Insertion and deletion	Albino	Feng et al., 2016
Maize	*Emp10*	Related to rice grain development	CRISPR-Cas9 knockout vector	Gene knockout	The grains are poorly developed	Cai, 2019
Single gene multiple site knockout						
Tomato	*SlAP3*	A direct homolog of *Arabidopsis* floral organ development gene (*AP3*)	CRISPR-Cas9 double site knockout vector	Deletion	Male sterility	Liu et al., 2019
Soybean	*GmFT2a*	Integrons in the photoperiodic flowering pathway	CRISPR-Cas9 knockout vector	Insertion and deletion	Late flowering	Cai et al., 2018
Soybean	*GmSPL3*	Regulate soybean plant morphology	CRISPR-Cas9 four site knockout vector	Insertion or deletion causes an early termination of the translation	Leaf size smaller, node number reduced, node spacing shortened, and plant height decreased	Wu et al., 2019
Multiple gene co-knockout						
Rice	*DEP1*	Rice spike-type-related gene	CRISPR-Cas9 co-knockout vector	Gene co-knockout (mutants with various combinations of mutations)	Erect spike, increase or decrease in grain size of spike	Shen et al., 2017
	*EP3*				Large spike, Grain number per spike increased	
	*Gn1a*				Grain number per spike increased	
	*GS3*	Rice grain-type-related gene			Increase in grain length and 1,000-grain weight	
	*GW2*				Grain width and yield increased	
	*LPA1*	Rice plant-type-related gene			Plant type loose	
	*BADH2*	Rice fragrance gene			The grain has a unique fragrance	
	*Hd1*	Rice photoperiod-related gene			Show a delayed heading stage under short-day conditions	
Rapeseed	Two *BnaMAX1* homologous genes	Regulation of plant height and axillary bud growth	CRISPR-Cas9 co-knockout vector	Insertion, deletion, or substitution	Semi-dwarfing, branching increase and yield increase	Zheng et al., 2020
*Brassica napus*	*BnaBRC1*, *BnaBRC2*	Related to rice branch	CRISPR-Cas9 co-knockout vector	Gene knockout	Distinct multiple branches	Amoo, 2020
Wheat	*TaAQ*, *T aDq*	Not given	CRISPR-Cas9 co-knockout vector	Deletion	Changes in plant height of *TaDq* deficient plants, *TaAQ* knockout plants were more fragile, and *TaAQ* and *TaDq* co-knockout plants were more brittle	Liu et al., 2020a
Gene knock-in						
Rice	*PPO1*	Herbicide resistance gene	CRISPR-Cas9 co-knock-up vector	Gene knock-up (chromosome fragment inversion)	Anti-herbicide	Lu et al., 2021
	*HPPD*	Herbicide resistance gene		Gene knock-up (chromosomal fragment duplication)		
Rice	Two genomic safe harbors	Synthetic carotenoids	CRISPR-Cas9 knock-in vector	Gene knock-in	Increased content of carotenoids	Dong et al., 2020
Gene regulation						
*Arabidopsis*	*AtFIS2*	*Arabidopsis* fertilization-independent seed2	CRISPR-dCas9-VP64 (transcription activator) vector	Gene silencing by demethylation of site C in the promoter CpG is lifted	Activated gene expression	Lowder et al., 2015
Strawberry	The uORF of *FvebZIPs1.1*	Encoding the basic (region) leucine zipper proteins	Base editor A3A-PBE-expression vector	Substitution	Strawberry sweetness increased	Xing et al., 2020
Rice	The 5′UTR intronic splicing site (5′UISS) of *Wx^a^*	Wx^a^ encoding a granule bound NDP-glucose-starch glucosyltransferase	CRISPR-dCas9 vector	Insertion and deletion	Different starch content	Zeng et al., 2020

### Application in Gene Knockout

Gene knockout is a key technology to study gene function and permanently change cell phenotype. CRISPR-Cas9 is applied to crops in a variety of ways. The traditional method is to construct a knockout vector, which is transferred into plants through agrobacterium-mediated transformation and other methods. The target gene knockout mutant can be obtained after 1–2 generations (Peng, 2017; Jiang et al., 2020). The target gene for gene knockout can be single or multiple. For each target gene, one or more sgRNAs can be designed to locate different target sites. Multiple sgRNAs can be assembled into a binary carrier at the same time through Golden Gate, Gibson, or isocaudomer technology (Arturo et al., 2015; Ma, 2015). Generally speaking, designing multiple targets for a gene will increase the efficiency of gene editing (Shi et al., 2018). It is relatively difficult to obtain co-knockout mutants of multiple genes in theory, but studies have shown that the tandem of multiple sgRNAs does not seem to affect editing efficiency (Shen et al., 2017).

#### Single-Gene One-Site Knockout

Liu et al. (2020b) used CRISPR-Cas9 to target and mutate the transcription factor *SlLBD40* in the border domain of tomato lateral organs to enhance the drought tolerance of tomato. Feng et al. (2016) used CRISPR-Cas9 technology for targeted knockout of maize *Zmzb7* gene, and successfully obtained the albino phenotype mutant material. In addition, based on the CRISPR-Cas9 system, they compared the gene mutation efficiency of the heterochromatic region and the homochromatin region of maize, and found that the mutation efficiency is around 50%. It shows that the gene targeting of the CRISPR-Cas9 system has nothing to do with chromatin state. Using CRISPR-Cas9 technology, Cai (2019) constructed knockout vector targeting at maize grain development gene *Emp10*. The mutant exhibited stagnant embryo and endosperm development, empty and deflated kernels after maturity, embryo lethality, and inability to germinate. Researchers analyzed the mechanism of the *Emp10* gene and found that the deletion of this gene affects the assembly and activity of mitochondrial electron transport chain complex I, impairs the oxidative phosphorylation pathway, affects ATP synthesis after fertilization, and leads to poor grain development.

#### Single-Gene Multiple-Site Knockout

Liu et al. (2019) constructed CRISPR-Cas9 expression vector targeting *SlAP3* gene. Sequencing results showed that deletion mutations occurred at 1–9 bp bases upstream of two target sites of *SlAP3* gene, resulting in early termination of *SlAP3* protein sequence translation. Thereby obtain male sterile tomato lines, homozygous mutants all showed phenotype of reduced number of flower organs such as petals and stamens. Cai et al. (2018) used CRISPR-Cas9 to induce targeted mutations of *GmFT2a* gene in soybean photoperiodic flowering pathway, and designed a total of three target sites, resulting in six frameshift mutations that all lead to early termination of translation, and all of the mutants showed late flowering phenotypes. Aiming at four target sites of *GmSPL3* gene, Wu et al. (2019) constructed CRISPR-Cas9 four-site knockout vector and successfully obtained spl3abcd mutants. Under short-day conditions, *spl3 abcd* mutants showed phenotypes of smaller leaves, fewer node, shortened polarity, and reduced height, indicating that *GmSPL3* plays an important role in regulating soybean plant morphology.

#### Multiple-Gene Co-knockout

In order to figure out the relevancy between different genes or obtain a plurality of excellent traits coexisting strains, the CRISPR-Cas9 polygenic knockout system is used to simultaneously knock out multiple genes. This makes it possible for obtaining strains with different combinations of good traits, providing new materials for crop breeding.

Shen et al. (2017) successfully constructed co-knockout vectors of eight genes related to agronomic traits in rice using CRISPR-Cas9 technology, and found that five of them (*EP3*, *GS3*, *GW2*, *DEP1*, and *Gn1a*) were related to yield, while the other three were related to flavor (*BADH2*), photoperiod (*Hd1*), and plant shape (*LPA1*). By screening the transgenic plants, various combinations of mutant strains are obtained, including homozygous mutants with a common mutation in six, seven, and eight genes, providing strategies for the rapid introduction of genetic diversity in crop breeding. The mutation efficiency of eight genes was found to be almost the same in the transient expression system with single-gene and multiple-gene knockout, suggesting that the number of sgRNAs in tandem may not affect the editing efficiency. Zheng et al. (2020) knocked out two *BnaMAX1* homologs in rapeseed by using targeted mutagenesis of CRISPR-Cas9 and obtain semi-dwarfed rapeseed mutants with increased branching and yield, the mutation rate of which was 56.3–67.38%. Two branching-related genes *BnaBRC1* and *BnaBRC2* in *Brassica napus* were mutated by using CRISPR-Cas9 technique, and the functional deletion mutant brc1 displayed an obvious multi-branch phenotype, indicating that BnaBRC1 is involved in the regulation of *Brassica napus* branch (Amoo, 2020). Liu et al. (2020a) used CRISPR-SpCas9 to edit *TaAQ* and *TaDq* genes in wheat. In T1 generation, *TaDq* deletion plants and wild-type (WT) plants were only different in plant height. In addition, *TaAQ* knockout plants or *TaAQ* and *TaDq* co-knockout plants were more vulnerable than WT plants.

### Application of Gene Knock-in

The most traditional application of CRISPR-Cas9 gene knockout can only reduce the expression level of genes, but a large number of genes can be improved by increasing their expression. To achieve the aim of it, gene knock-in or knock-up might be an efficient approach.

A general technical route for knock-up is to insert regulatory elements such as promoters or enhancers near the target gene. The herbicide resistance of rice can be improved by transgenic overexpression of *PPO1* and *HPPD*, which are two herbicide resistance genes in rice. However, there is randomness in the integration of target genes into the genome in traditional transgenic technology. Linjian Jiang’s team improved the herbicide resistance of rice through CRISPR-Cas9 double-target editing. The first target is the highly expressed gene *CP12* near *PPO1* and its promoter, and *PPO1* gene was driven by the promoter of *CP12* by fragment inversion. The second target is the highly expressed gene *Ubiquitin2* near *HPPD* and its promoter, and the high expression of *HPPD* could be drove by the promoter of Ubiquitin2 through segment repetition (Lu et al., 2021).

Inserting the target gene into the appropriate genome site is an effective approach to get gene knocked-in. The traditional random integration of vectors into plant genome based on agrobacterium or particle bombardment sometimes reduces the crop yield. But CRISPR-Cas9 technology can be used to achieve targeted insertion at genome safe harbor (GSHs), thus avoiding the impact on crop yield. GSH is the region in chromosome that contains transgenes, which will not adversely affect the host organism due to genome destruction. Dong et al. (2020) used CRISPR-Cas9 technology to insert a 5.2 kb carotenoid biosynthetic cassette in rice genome safe harbor, and successfully obtained unmarked rice plants with high carotenoid content in seeds.

### Applications in Gene Regulation

According to the process of gene expression, gene regulation can be divided into transcriptional regulation and post-transcriptional regulation, and transcriptional regulation can be divided into DNA regulation at the genetic level and chromatin regulation at the epigenetic level (Zhu et al., 2019). As one of the most flexible systems in genome regulation technology, CRISPR-Cas9 functions mainly through DNA regulation at genetic level.

By modifying the Cas protein, a protein with loss of nuclease activity and retention of DNA sequence recognition, namely dead Cas9 (dCas), was obtained (Liu et al., 2020c). The binding characteristics of dCas with double-strand target genes were similar to those of transcription factors regulating gene expression, but dCas cannot function alone. Therefore, researchers combined dCas with transcriptional activator protein/transcriptional suppressor protein to activate or inhibit gene transcription (Luo and Gu, 2016). In *Nicotiana benthamiana* leaves, dCas9 fused to the transcriptional activator EDLL controls the expression of different genes in leaves (Piatek et al., 2015).

Targeting sgRNA in promoter region can regulate gene expression more efficiently (Moradpour and Abdulah, 2020). For example, the transcriptional activator VP64 was fused with dCas9 and bound to C of CpG methylation site in *Arabidopsis thaliana* promoter region, which relieved the inhibition of CpG methylation on *AtFIS2* gene transcription (Lowder et al., 2015). In addition, alleles with different transcriptional and phenotypic characteristics were obtained by using eight gRNAs targeting cis-regulatory elements (CREs) in the CLV3 promoter region of tomato (Rodríguez-Leal et al., 2017).

The researchers also targeted sgRNA to open reading frames and splicing-related sites. The uORF of diploid strawberry *bZIP1.1* was edited by cytosine base editor (A3A-PBE), which improved the translation of primary ORF and increased sweetness in strawberries (Xing et al., 2020). Zeng et al. (2020) used CRISPR-dCas9 system to target the 5′ UTR intron splicing site (5′ UISS) of *Wx^a^* gene, which controls starch synthesis, for post-transcriptional regulation to obtain mutants with different starch content, providing a new method for cultivating high-quality rice.

In addition, although dCas cannot play a role alone, it can cause steric hindrance. When the DNA sequence bound by dCas is the promoter or transcription start site of the target gene, it can prevent the initiation of transcription; when dCas binds to the reading frame of the target gene, it can prevent RNA polymerase binding, transcription factor binding, and transcription extension (Moradpour and Abdulah, 2020; Guan et al., 2021).

Besides, CRISPR-Cas9 also plays a role through epigenetic chromatin regulation, using dCas9 to bind DNA methylase and acetylase to modify genes, or changing chromatin structure to regulate gene expression by changing the interaction between enhancer and promoter (Moradpour and Abdulah, 2020; Zhu et al., 2020).

## The Applications of CRISPR-Cas9 Technology in Crops

Researchers are paying attention to CRISPR-Cas9 because of its high efficiency. A large number of experiments have proved that this technology is suitable for a variety of crops, such as rice, wheat, maize, soybean, and potato, and can achieve the expected experimental results, among which, rice is the most widely used. Crop improvement is usually carried out by increasing crop yield, improving crop quality, obtaining biological and abiotic resistance, and obtaining male sterile materials. Table 2 summarizes the application of CRISPR-Cas9 in the crop improvement of rice, wheat, maize, soybean, and potato.

**Table 2:** Applications of CRISPR-Cas9 technology in crops.

Species	Target gene	Gene function	CRISPR-Cas technical principles	Mutation mode	Mutant features	References
Rice						
	*OsSLR1*	Encoding DELLA protein, inhibit gibberellin synthesis and seed germination	CRISPR-Cas9 three-site knockout vector	Gene knockout	The germination rate was increased	Geng et al., 2021
	*TGW6*	1,000-grain weight related gene in rice	CRISPR-Cas9 three-site knockout vector	Insertion or deletion	1,000 grain weight increased by 5%	Wang et al., 2016a
	*OsFWL2*	Gene regulating grain number per spike in rice	CRISPR-Cas9 double-site knockout vector	Insertion or deletion	Spike number and single-strain yield were increased	Wang, 2018
	*Hd2/Os PRR37*	Main effect gene in rice heading stage	CRISPR-Cas9 co-knockout vector	Co-knockout (base substitution, insertion or deletion)	Precocious, scented rice	Zhou, 2017
	*Hd5/DTH8/Ghd8*					
	*Hd4/Ghd7*					
	*Badh2*	Rice fragrance gene				
	*Wx*	Negative regulation of amylose content	CRISPR-Cas9 knockout vector	Gene knock-out	Amylose content was significantly reduced in non-waxy sterile lines	Feng et al., 2018
	*OsAAP6*, *OsAAP10*	Amino acid transporter genes	Construct CRISPR-Cas9 knockout vectors respectively	Single-base insertions or deletions, and large fragment were absent	Glutamate content, high grain protein content were all decreased, and amylose content were significantly reduced	Wang et al., 2020c
	*Pita*	Negative regulating the resistance of rice blast	CRISPR-Cas9 co-knockout vector	Insertion and deletion	The resistance to rice blast was increased, and the expression of genes associated with the signal transduction pathway such as salicylic acid, jasmonic acid, and ethylene was upregulated in the homozygous mutant strains	Xu et al., 2019
	*Pi21*	Recessive rice blast resistance gene		Insertion, deletion, and substitution		
	*ERF922*	Negative regulating the resistance of rice blast		Insertion, deletion, and substitution		
	*HW3*	Susceptibility gene of bacterial blight of rice	CRISPR-Cas9 knockout vector	Gene knockout	Enhanced resistance to bacterial blight of rice	Hao, 2016
	*Os8N3*	Encodes a member of the sugar transporter family, and is a Xanthomonas susceptibility gene	CRISPR-Cas9 knockout vector	Gene knock-out	Sucrose concentration in embryo sac decreased, grain filling defects, resistance to Xanthomonas	Kim et al., 2019
	*AFP1*	Abiotic stress related gene in rice	CRISPR-Cas9 double-site knockout vector	Small fragments deletion, and substitution	The ability of cold resistance, heat resistance, and osmotic stress was improved	Zhou et al., 2021b
	*OsALS*	Encodes acetolactate synthase	CRISPR-Cas9 knockout vector	Substitution	Plant height became shorter and herbicide resistance increased	Wang et al., 2020a
			CBE	Base substitution (C-T)	Tolerant to IMI herbicide	Wang et al., 2019
	*OsNramp5*	Key ion transporters of Mn, Cd, and Fe absorption in rice roots	CRISPR-Cas9 knockout vector	Gene knock-out	Low cadmium	Long et al., 2019
	*PTGMS2-1*	Male fertility genes	CRISPR-Cas9 knockout vector	Insertion and deletion	Photoperiod/heat sensitive male sterility	Lan et al., 2019
	*TMS5*	Temperature sensitive male sterility gene	CRISPR-Cas9 knockout vector	Insertion and deletion	Thermosensitive male sterile rice, the starting temperature of thermosensitive sterility of T2 generation TMS lines was about 24°C	Wu et al., 2018
	*ZEP1*	Encoding the central element of the meiotic association complex	CRISPR-Cas9 knockout vector	Gene knockout	Male sterility, female fertility is normal, genetic recombination efficiency was greatly increased, and genetic interference was completely eliminated	Liu et al., 2021a
Wheat						
	TaGASR7	Negative regulation of grain length and grain weight genes	Transient expression of CRISPR-Cas9 DNA or RNA	Insertion and deletion	1,000 grain weight increased	Zhang et al., 2016	
	Wheat-glial gene	Genes associated with glutelin synthesis	CRISPR-Cas9 knockout vector	Insertion and deletion	Low in glutelin, non-transgenic wheat	Sánchez-León et al., 2017
	*Ta SBEIIa*	Genes related to starch synthesis in wheat	CBE based on CRISPR-Cas9	C-T single base substitution	Nutrition content such as amylose, resistant starch, protein, and soluble pentosan were significantly improved	Li, 2021a
	*MLO*	The powdery mildew susceptibility gene	CRISPR-Cas9 knockout vector	Insertion	Resistance to powdery mildew	Wang et al., 2014
	Three homologs of *EDR1*	A negative role in the defense response to powdery mildew	CRISPR-Cas9 knockout vector	Gene knock-out	Resistance to powdery mildew	Zhang et al., 2017
	Three *TaNP1* homologous alleles	Probably encoding a glucose-methanol-choline oxidoreductase, it is necessary for male sterility	CRISPR-Cas9 knockout vector	Triple gene knockout	Triple homozygous mutants were completely male sterile	Li, et al., 2020b
Soybean												
	*GmNARK*	A receptor kinase that inhibits the expression of nodule formation related genes	CRISPR-Cas9 three-site knockout vector	Insertion and deletion, resulting in early termination of translation	Supernodules, short plants, and dark green leaves	Bai et al., 2019
	*GmFAD2-1A*	Key enzyme that catalyze the conversion of oleic acid to linoleic acid	CRISPR-Cas9 three-site knockout vector	Deletion and substitution	The oleic acid content was significantly improved	Hou et al., 2019
	*GmFATB1a*, *GmFATB1b*	Encoding a thioesterase, is a key enzyme for fatty acid synthesis	CRISPR-Cas9 co-knockout vector	Deletion	The content of saturated fatty acid was reduced and the double mutants showed male sterility	Jing et al., 2021
	*GmSnRK1.1*, *GmSnRK1.2*	Participate in anti-stress pathways	CRISPR-Cas9 co-knockout vector	Deletion	Reduced sensitivity to abscisic acid	Li et al., 2018
Maize												
	*LIG*	Liguleless1	(1) CRISPR-Cas9 knockout vector (2) SNP based on CRISPR-Cas9	Insertion	Not given	Svitashev et al., 2015, 2016
	*MS26*	Male fertility genes		Insertion and deletion	Not given		
	*MS45*			Insertion and deletion	Not given		
	*ALS1*	Encodes acetolactate synthase		Insertion and deletion	Not given		
	*ALS2*			Insertion and deletion	Resistant to chlorsulfuron		
	The 5′ untranslated section of *ARGOS8*	Negative regulator of ethylene reaction	CRISPR-Cas9 knock-in vector	Promoter insertion	Yields increase under drought conditions	Shi et al., 2017
	*ZmBADH2-1*, *ZmBADH2-2*	Homologous genes that control fragrance genes	CRISPR-Cas9 co-knockout vector	Insertion, deletion, and substitution	The grains have a fragrant rice flavor	Zhang et al., 2017
	*MS8*	Male fertility genes	CRISPR-Cas9 knockout vector	Substitution	Male sterile	Chen et al., 2018
Potato												
	*GBSS*	Encoding granular bound starch synthase	CRISPR-Cas9 four-site knockout vector	Insertion and deletion	Amylose content increased significantly in homozygous mutant plants	Andersson et al., 2017
	*StSSR2*	Encoding the sterol side chain reductase 2 genes, a key gene for the synthesis of solanine	CRISPR-Cas9 co-knockout vector	Insertion, deletion, and substitution	Low solanine, low temperature glycosylation resistance, and high amylopectin	Zhao, 2019
	*VInv*	Acid vacuolar invertase that plays a key role in low temperature saccharification					
	*GBSSI*	Grain binding starch synthase gene associated with amylose synthesis					
	*S-RNase*	Participates in the control of gametophyte self-incompatibility	CRISPR-Cas9 knockout vector	Early termination of translation	Self-compatible diploid lines of potato	Enciso-Rodriguez et al., 2019

### Rice

As one of the most important food crops, rice is the staple food for more than half of the world’s population. It is expected that by 2030, Chinese population will reach 1.65 billion, with a food deficit of 140 million tons. It is urgent to improve rice yield and crop quality (Guo et al., 2019). As an efficient genome editing technology, CRISPR-Cas9 has made great contributions to rice improvement in recent years.

Rice germination rate, tiller number, panicle number per plant, grain number per panicle, and thousand grain weight are the key indicators to determine rice yield, which can be improved by editing-related genes. Gibberellin (GA) is a promoter of seed germination, which can relieve seed dormancy and stimulate germination, while DELLA protein is a key protein that inhibits the signaling pathway of GA. SLR1 is the DELLA protein in rice. Geng et al. (2021) used CRISPR-Cas9 gene editing technology to construct a rice *OsSLR1* knockout vector. Compared with wild-type Nipponbare rice, the *slr1* mutant has a significantly higher germination rate and faster growth rate 2 days after sowing. In order to solve the problem of uneven germination rate during rice planting, grain weight of rice is one of the three major factors affecting rice yield. Grain weight is determined by the synthesis and accumulation of starch in grains and the size of grains. It is a typical quantitative trait. *TGW6* gene is one of rice grain weight-related genes, encoding an indoleacetic acid-glucose hydrolase. The loss-of-function mutant can cause a decrease in the content of indoleacetic acid in the endosperm, thereby increasing the cell number, grain length, and grain weight, and carbohydrate accumulation before heading, thereby increasing rice yield. Wang et al. (2016a) took rice *TGW6* as the target gene and carried out three-point mutation through CRISPR-Cas9 technology. The mutation rate of T0 generation was 90%, and the thousand-grain weight of mutant offspring increased by 5%, laying the foundation for the cultivation of high-yield rice. The *FWL2* gene is widespread in plants and controls organ and fruit size by inhibiting cell division. Wang (2018) used CRISPR-Cas9 technology to design two target sites for *OsFWL2*, a gene that regulates grain number per panicle in Zhonghua 11, to construct knockout vectors, respectively, creating lines with significantly increased grain number per panicle and yield per plant, providing new germplasm for rice yield improvement.

Clustered regularly interspaced short palindromic repeats-Cas9 technology is used to increase the aroma of rice and control the synthesis of amylose and glutamate to improve the edible quality of crops. The main component of rice aroma is 2-acetyl-1-pyrroline (2-AP). *Badh2*, a recessive gene that controls aroma in rice, encodes an aldehyde dehydrogenase that oxidatively inactivates 2-AP synthesis precursors (Ren et al., 2021). Qi et al. (2020) used the CRISPR-Cas9 system to edit *OsBadh2* of Nipponbare, and successfully obtained rice mutants with increased 2-AP content and significantly improved aroma traits. Amylose content in rice is a key component affecting edible quality of rice. Rice *Waxy* genes (*Wx*) are the main genes controlling amylose content in rice. Granule-bound starch synthaseI (GBSSI) encoded by *Wx* gene directly controls synthesis of grain amylose. Feng et al. (2018) used Liu Yaoguang’s multi-target intelligent editing system to edit the Wx locus in the rice excellent maintainer line 209B, and obtained the glutinous rice maintainer line WX209B with an amylose content of 1%, which was then transformed into the glutinous rice sterile line WX209A. The homozygous deletion mutation rate in the T0 generation was 26.9%, proving that the multi-target gene editing system can increase the homozygous mutation rate, and homozygous mutants can be stably inherited to offspring. The *AAP* gene family encodes amino acid permease. *AAP6* gene can not only enhance the expression of key genes of storage proteins, but also promote the absorption of amino acids by plants and increase the protein content. High grain protein content (GPC) decreases eating and cooking quality (ECQ) of rice. Using the CRISPR-Cas9 system to mutagenize the amino acid transporter genes *OsAAP6* and *OsAAP10*, in four varieties with different genetic backgrounds, the content of glutamate protein can be reduced, thereby improving the quality of rice. The GPC of T1 generation decreased by 2.9–19.1%, and the GPC of T2 generation decreased by 1.5–10.4% (Wang et al., 2020c).

Rice blast and bacterial blight of rice are two major diseases leading to rice yield reduction, and CRISPR-Cas9 was used to obtain disease-resistant rice by editing susceptible genes. The *OsERF922* gene encodes an AP2/ERF-like transcription factor that is strongly induced by both pathogenic and non-pathogenic rice blast fungi and negatively regulates rice blast resistance. Wang et al. (2016b) used CRISPR-Cas9 to target and mutate the rice ERF transcription factor gene *OsERF922* to enhance rice blast resistance and obtain genetic materials without changes in main agronomic traits, providing new germplasm resources for rice breeding. The *Pi21* gene is an invisible rice blast resistance locus, and its expression product is rich in proline. The reason of disease resistance is that there is a small fragment deletion in the proline region. The substitution of 918 amino acids of the protein encoded by the *Pita* gene leads to rice blast resistance. Xu et al. (2019) used CRISPR-Cas9 technology to construct a co-editing vector with *Pita*, *Pi21*, and *ERF922* as target genes. The triple mutant homozygous line with enhanced rice blast resistance was successfully obtained, which has high utilization value. Rice bacterial blight caused by Xanthomonas oryzae pv. Oryzae (Xoo) leads to a 50% reduction in rice yield. Hao (2016) used CRISPR-Cas9 technology to identify the bacterial blight susceptibility-related gene *HW3* in rice. The first exon was modified by site-directed modification, and rice blast resistant plants were successfully obtained. The results of field inoculation phenotype showed that it inhibited the early development of pathogen Xoo, but after inoculation with pathogen 15, the lesions appeared to grow, so it was not the main susceptibility genes. The pathogenicity of Xoo relies on a class of effectors called transcription activator-like (TAL) effectors, *Os8N3* gene belongs to the sugar final export sugar transporter (sweet) family and is one of the susceptibility genes induced by TAL effectors. Os8N3 can scavenge toxic copper in xylem vessels where Xoo proliferates and spread, and makes Xoo readily available nutrients for its growth and toxicity to cause disease. Kim et al. (2019) used CRISPR-Cas9 technology to target mutant rice *Os8N3*. A mutant strain resistant to Xanthomonas oryzae was obtained, and agronomic traits including pollen fertility were hardly affected, indicating that this method can be successfully used for crop breeding.

Drought, salt, weeds, and heavy metals have always been the resistance to the growth and development of rice. CRISPR-Cas9 has great significance in improving the abiotic resistance of rice. Due to the global climate change, the frequency of drought is increasing, and drought has become an important abiotic stress of rice yield fluctuation (Rao et al., 2020). Zhou et al. (2021b) used CRISPR-Cas9 technology to knock out the *AFP1* gene to improve the drought tolerance, heat tolerance, and osmotic stress ability of rice. On the one hand, the root length was significantly increased, and on the other hand, the sensitivity to abscisic acid and the water loss rate of leaves were reduced. Other agronomic traits changed, plant height and seed setting rate decreased, effective tillering increased, panicle length increased significantly, and yield per plant varied between 4.06 and 11.75%. The *OsALS* gene encodes acetolactate synthase, and existing studies have shown that mutations in the *ALS* gene are tolerant to the herbicide imidazolinone (IMI). Current studies have shown that loss-of-function mutations in *ALS* genes can be lethal, so it is very important to obtain non-function-free genes with *als* mutations. Wang et al. (2019) optimized the CBE system to obtain base editors with improved editing efficiency. Successful substitution of C-T at position 1882 of the *ALS* gene, the mutation efficiency is as high as 71.4%, and the mutant strain is tolerant to IMI herbicide. Wang et al. (2020a) took the acetolactate synthase (*OsALS*) gene as the target and used CRISPR-Cas9 technology to mutate *OsALS*, the C-T substitution at nucleotide 1882 to form a new rice herbicide resistance allele *G628W*, increased the herbicide resistance of rice, and the feasibility of creating new genetic variation for crop breeding. The non-transgenic progeny with the homologous *G628W* allele exhibited similar agronomic characteristics to wild-type plants in addition to reduced plant height. Rice is a cadmium-rich plant. Excessive intake of cadmium will seriously endanger human health. The *OsNramp5* gene encodes a manganese and iron transporter, which is the main protein for cadmium absorption in roots, and its loss can significantly reduce the content of cadmium in grains. Long et al. (2019) constructed a CRISPR-Cas9 knockout vector for *OsNramp5* gene, and successfully created low-cadmium indica rice. The cadmium content of four *OsNramp5* gene knockout lines grown under non-cadmium pollution conditions was lower than 0.02 mg/kg, which was on average higher than that of wild-type. When planted in cadmium-contaminated soil, the grain cadmium content of *OsNramp5* knockout lines of different varieties was lower than 0.1 mg/kg, an average reduction of 94.8% compared with wild-type.

Male sterile lines are the core of two-line hybrid rice breeding. Traditional hybrid breeding methods have long breeding cycle and high labor intensity. Non-transgenic male sterile lines can be obtained by using CRISPR-Cas9 technology to knock out rice fertility genes and after 1–2 generations of breeding, providing excellent materials for rice breeding (Wu et al., 2018). Lan et al. (2019) took 93–11 and Huazhan’s male fertility gene *PTGMS2-1* gene as the target gene, used CRISPR-Cas9 technology to construct an expression vector, and successfully obtained photoperiod/heat-sensitive gene male sterile rice. Almost completely sterile, with increased tiller number and significantly lower plant height compared to WT, with basically normal agronomic traits. Using rice *TMS5* as the target gene, Wu et al. (2018) constructed the CRISPR-Cas9 gene editing vector, transformed the excellent intermediate material GH89, and successfully obtained thermo-sensitive two-line sterile plants. Liu et al. (2021a) constructed the *ZEP1* gene knockout vector (encoding the central element of meiosis synapsis complex) in rice using CRISPR-Cas9 technology, and obtained mutant zep1 with the male sterile and female fertile. Genetic analysis showed that in the absence of *ZEP1*, the efficiency of genetic recombination was greatly increased, and the genetic interference was completely eliminated. These results indicated that synapsis played a role in regulating rice disturbance. At the same time, the successful acquisition of this mutant provides a new method for increasing genetic diversity in crop breeding.

### Wheat

Wheat is the second largest food crop in the world. Increasing wheat production is also an important way to alleviate population growth and environmental degradation. Wheat is allohexaploid with a huge genome, and this characteristic increases the difficulty of breeding. But the high specificity and high efficiency of the CRISPR-Cas9 system reduces the difficulty of this. In recent years, researchers have obtained stable inherited mutants with excellent traits, applying CRISPR-Cas9 technology to wheat (Wang et al., 2014).

Similar to cereal crop rice, genes related to thousand-grain weight of wheat are the key to affecting wheat yield. Zhang et al. (2016) used *TaGASR7* as the target gene to bombard the callus of hexaploid bread wheat and tetraploid durum wheat through transient expression of CRISPR-Cas9 DNA or RNA, and obtained T0 mutants with increased thousand-grain weight.

High-glutelin wheat can cause diseases such as celiac disease in susceptible people. Editing genes related to glutelin synthesis is an effective way to reduce glutelin content. Based on CRISPR-Cas9 technology, Sánchez-León et al. (2017) constructed pANIC-CR-*Alpha1* and pANIC-CR-*Alpha2* expression vectors by using the conserved region near the coding sequence of the immune dominant epitope of wheat glutelin as target sites, they successfully obtained low-protein, non-transgenic wheat through transformation. Resistant starch is a kind of starch that is difficult to digest, absorb, and enter the bloodstream. It has the functions of controlling weight, lowering blood sugar, and promoting the absorption of vitamins and minerals (Yang et al., 2020). *SBE* is a key gene for amylopectin synthesis. The synthetic pathway of resistant starch is similar to that of amylose, and there is a significant positive correlation. Inhibiting the expression of *SBE* gene will increase the synthesis of resistant starch. Using CRISPR-Cas9 technology to construct rice gene C-T single base editing vector, the starch branching enzyme gene *TaSBEIIa* was knocked out, and a new high-resistant starch winter wheat germplasm was obtained (Li, 2021a).

Powdery mildew is a major fungal disease of wheat crops, causing severe yield losses in wheat. Mildew resistance locus O (*MLO*) genes are plant-specific family genes, and *mlo* mutants have broad resistance to powdery mildew fungi. Wang et al. (2014) used the CRISPR-Cas9 system to realize the directed mutation of the wheat powdery mildew susceptibility gene *MLO*, and successfully created wheat with long-lasting and broad-spectrum resistance to powdery mildew, which will provide an important starting material for the disease resistance breeding of wheat powdery mildew. Zhang et al. (2017) used CRISPR-Cas9 technology to simultaneously modify three homologs of the wheat *EDR1* (enhanced disease resistance1) gene to obtain a mutant strain resistant to powdery mildew without mold-induced cell death. Therefore, this potentially valuable traits generated by CRISPR-Cas9 technology may provide new germplasm for disease resistance breeding.

In hybrid seed production, the male sterile line of wheat can be used as the female parent to improve the seed purity of excellent offspring and overcome the difficulty of artificial pollination. Wheat is allohexaploid, so it is difficult to obtain nuclear recessive male sterile mutants with traditional methods. The CRISPR-Cas9 system can realize the simultaneous mutation of multiple homologous alleles and obtain male sterile plants (Sun, 2019; Li et al., 2020b). The homologous genes of *TaNP1* gene are *OsNP1* gene and *ZmIPE1* gene in rice and maize, respectively, both of which encode a glucose-methanol-choline oxidoreductase. The *osnp1* mutant and *zmipe1* mutant showed complete male sterility. Li et al. (2020b) used CRISPR-Cas9 technology to edit three *TaNP1* homologous alleles to obtain a completely male sterile *TaNP1* triple homozygous mutant wheat.

### Soybean

Soybean is an important oil crop and the main source of human plant protein, which has important economic value. With the increase in the world’s population and the improvement of human living standards, it is of great significance to use CRISPR-Cas9 technology to quickly cultivate high-yield and high-quality soybean varieties.

Rhizobium is a symbiosis of legumes and rhizobia, which can fix nitrogen in the atmosphere and provide nitrogen nutrition for plant growth. The biological nitrogen fixation of soybeans provides an important guarantee for the sustainable development of agriculture. However, nitrogen fixation is an energy-consuming process, too many nodules will become a burden on soybeans, so the number of nodules is regulated by the nodulation autonomous regulatory mechanism. *GmNARK* gene encodes a receptor kinase in the regulatory pathway. Using CRISPR-Cas9 technology, three sgRNAs were designed to knock out the *GmNARK* gene, and the mutants with super nodulation, short plant and dark green leaf phenotype were successfully obtained, which provided genetic material for studying the mechanism of nodulation and nitrogen fixation in soybean, and provide new germplasm resources for soybean molecular breeding (Bai et al., 2019).

Using CRISPR-Cas9 technology to knock out genes related to the synthesis of soybean protein, oleic acid, and saturated fatty acids is the key to obtaining high-quality soybean oil (Cai, 2016). Oleic acid is an unsaturated fatty acid, which has the effect of lowering cholesterol, slowing down atherosclerosis and preventing cardiovascular disease, so cultivating soybeans with high oleic acid content has a very important use value. Delta-twelve fatty acid desaturase 2 enzyme (FAD2) is a key enzyme that catalyzes the formation of linoleic acid from oleic acid. Inhibiting the *FAD2-1A* gene can increase the ratio of oleic acid/linoleic acid in soybean seeds. Targeting the soybean oleic acid gene *GmFAD2-1A*, Hou et al. (2019) constructed a CRISPR-Cas9 editing vector fused with three gRNAs, and increased the oleic acid content in soybean seeds of Huaxia 3 from 20 to 23%, without significantly affecting agronomic traits including total protein and total fat content in soybean seeds. Excessive intake of saturated fatty acids in humans can cause elevated cholesterol levels and increase the risk of atherosclerosis. FATB protein is a thioesterase that has the function of releasing free fatty acids and ACP, and the released fatty acids are involved in fatty acid chain elongation. Jing et al. (2021) used CRISPR-Cas9 technology to knock out the *GmFATB1* gene encoding the FATB protein, which significantly reduced the content of two saturated fatty acids in soybean seeds, providing excellent materials for soybean breeding.

Abiotic stress is an important factor affecting soybean yield, and it is of great significance to knock out soybean abiotic stress-sensitive genes. Sucrose non-fermenting related protein kinases (SnRKs) are a class of Ser/Thr protein kinases that are widely present in plants and play important regulatory roles in plant growth, development, metabolism, and stress resistance. *GmSnRK1.1* and *GmSnRK1.2* are two homologous genes of *SnRK1*. Li et al. (2018) used *GmSnRK1.1* and *GmSnRK1.2* as targets to construct a CRISPR-Cas9 double gene knockout vector. The double gene mutation rate was 48.6%, and mutants have reduced sensitivity to abscisic acid.

### Maize

Maize is the third largest cereal crop after rice and wheat with a wide planting area. New varieties of maize accounted for more than 35% in various factors of increasing yield. Compared with traditional breeding, CRISPR-Cas9 technology has strong specificity, short breeding cycle and other advantages, which greatly promotes the creation of excellent maize varieties (Zhu, 2015).

Clustered regularly interspaced short palindromic repeats-Cas9 technology is used to improve the abiotic stress resistance of maize. Svitashev et al. (2015) used CRISPR technology to construct an editing vector for the maize *ALS2* gene encoding acetolactate synthase and successfully obtained chlorsulfuron-resistant plants. Drought is an important factor affecting the yield of maize. Shi et al. (2017) used the CRISPR-Cas9 system to insert the GOS2 promoter into the 5′untranslated region of *ARGOS8* gene by targeting the promoter of *ARGOS8* gene, a negative regulator of ethylene reaction in maize, and obtained drought-resistant maize varieties with improved yield.

In the face of human demand for maize edible quality, CRISPR-Cas9 technology is used to edit maize flavor-related genes to obtain fragrance maize. *BADH2* gene encodes betaine aldehyde dehydrogenase. The mutation of *BADH2* can inhibit the synthesis of aroma substance 2-AP. *ZmBADH2-1* and *ZmBADH2-2* genes are the homologous genes of *BADH2* gene in maize. Zhang et al. (2021) constructed CRISPR-Cas9 co-knockout vectors of *ZmBADH2-1* and *ZmBADH2-2* genes, and successfully created maize varieties with fragrant rice flavor in grains. Whether the mutation of this gene affects other agronomic traits remains to be further studied.

Male sterile lines are important materials for the breeding of many crops. CRISPR-Cas9 technology has shown great potential in breeding male sterile lines. Svitashev et al. (2016) designed specific gRNAs based on the CRISPR-Cas9 system for maize liguleless1 (*LIG*), acetolactate synthase gene (*ALS2*), and two male fertility genes (*MS26* and *MS45*). Then, maize embryo cells were bombarded with particles by ribonucleo protein complex, which assembled from Cas protein and gRNA. Compared with DNA delivery experiments, it was found that the mutation frequency and mutation types of the two were similar, and the off-target rate of RNP delivery and the homozygous mutation rate was also significantly reduced. Chen et al. (2018) constructed a CRISPR-Cas9 vector for the maize MS8 gene and transformed it. The *MS8* gene was not detected in the T0 transgenic lines obtained, but the *MS8* gene could be detected in the F1 and F2 generations. They screened the male sterile plants. In the F2 generation, a transgenic-free *ms8* male sterile plant was successfully obtained, which can be used in a maize hybrid production system without being restricted by the regulatory framework of genetically modified organisms (GMO). In addition, there was an off-target site in this study, but it did not cause adverse effects on plants.

### Potato

Potato is an important food crop in China, not only as a vegetable consumption, but also an important raw material for starch production. However, due to the complex potato genetic background, self-incompatibility characteristics, and long traditional hybrid breeding cycle, low predictability, new variety breeding process is slow, using CRISPR-Cas9 technology can effectively accelerate the breeding process (Li, 2019).

Changes in the ratio of amylose and amylopectin in potatoes will greatly change the properties of starch. Changing the ratio of potato starch or containing only one starch will gain advantages in many applications (Zeeman et al., 2010). Andersson et al. (2017) targeted potato grain binding starch synthase (*GBSS*) gene and constructed targeted knockout vector using CRISPR-Cas9 system. The results showed that only when all four alleles of *GBSS* were mutated, the amylose content in the corresponding positive plants decreased significantly compared with the wild-type, while the amylopectin content increased. However, the homozygous mutant with increased amylopectin content also has the problem of unstable DNA integration and needs to obtain stable genetic offspring through hybridization. Zhao (2019) targeted three genes in potatoes: Sterol side chain reductase 2 gene (*St SSR2*), acid vacuole invertase gene (*VInv*), and grain binding starch synthetase gene (*GBSSI*), were simultaneously knocked out by CRISPR-Cas9 technique. A new potato strain with low solanine resistance, low-temperature saccharification resistance, and high amylopectin resistance were developed. The knockout of *St SSR2* has no effect on the phenotype and physiological indexes of potato. After the complete knockout of *VInv*, the reducing sugar content of potato decreases. After the knockout of *GBSSI* gene, the amylose content is completely suppressed, there was no obvious effect on the type, physiological index and tuber yield, and it was basically suitable for field cultivation.

The tetraploid character of potato can hinder the breeding process, so researchers tried to invent diploid inbred lines, but due to the diploid gametophyte self-incompatibility, prevented the production of diploid homozygous lines. The S locus RNase (*S-RNase*) gene is associated with gametophyte self-incompatibility, Enciso-Rodriguez et al. (2019) successfully obtained a stable self-compatible potato diploid lines by targeting *S-RNase* gene with CRISPR-Cas9 system, which greatly promoted potato breeding.

## Problems With CRISPR-Cas9

### Technical Problems

#### Off-Target Effect

The off-target effect of CRISPR-Cas9 technology is caused by random cutting of non-target sites in the genome by Cas9. There are two main reasons for the off-target effect.

Firstly, sgRNA may bind to untargeted sequences. The CRISPR-Cas9 gene editing system relies on the specific binding of the recognition sequence of sgRNA with the targeted sequence. However, due to the extreme complexity of the genome, sgRNA designed according to the target site may match with other sequences similar to the target sequence. This local match also activates Cas9 endonuclease activity, resulting in off-target effect.

Secondly, Cas9 may recognize non-standard PAM. CRISPR-Cas9 gene editing system relies on Cas9 to cut three bases upstream of PAM site. However, the actual situation is that Cas9 can not only recognize the standard PAM near the target site, but also recognize the non-standard PAM. If non-standard PAM is ignored when designing the targeted sequence, it may also cause a certain degree of off-target (Yuan et al., 2017).

#### Exogenous Genes

Editing vectors carrying Cas9 and sgRNA usually infect plants through agrobacterium-mediated transformation. Some vector fragments may be randomly integrated into the plant genome, thus introducing foreign genes. The existence of foreign components may cause unexpected biosafety problems (Bao et al., 2019). For sexually reproducing crops like rice, plants without exogenous components can be screened from progeny by genetic separation of sexual generations. However, crops that do not undergo sexual reproduction, such as potato, cannot isolate and remove foreign genes through genetic separation, so it is impossible to avoid the residue of foreign gene components (Yao et al., 2017).

Therefore, the elements of editing vector can be loaded in the form of ribonucleoprotein, which can be applied to crops without genetic separation of sexual generations to avoid the introduction of foreign genes at the beginning (Svitashev et al., 2016; Zhen et al., 2017). However, this method is difficult to construct and operate, so it needs to be optimized to improve its efficiency and adapt to more crops. He and Zhao (2020) introduced several methods for obtaining transgenic-free plants for asexual plants, such as nbonucIeoprotem transfection, transient expression of transgenes without DNA integration, and nano-biotechnology.

Recently, Liu et al. (2021b) developed a web tool for foreign element detection (FED) of genome-edited organism. It can complete the sequence detection of 46,695 exogenous components at one time according to the whole genome sequencing data when the information of exogenous vector components is unknown. Meanwhile, the FED can accurately identify the fragment length and insertion position of exogenous components in the genome. It provides an efficient tool for biosafety detection of genome editing products.

### Regulatory Issues

At present, the crop regulation problem caused by CRISPR-Cas9 technology is due to the introduction of foreign genes when CRISPR-Cas9 technology is applied in crops, resulting in unpredictable biosafety. From a scientific point of view, CRISPR-Cas9 technology realizes mutation through cell self-repair, which is very similar to natural mutation, physical mutagenesis, and chemical mutagenesis of crops, but the essence cannot be judged (Wang and Zhao, 2018). There are differences in the regulatory policies of CRISPR-Cas9 edited plants around the world.

Compared with the strict and clear regulatory policies for GMO, countries around the world lack clear and consistent supervision policies for CRISPR-Cas9 edited plants, and there are also disputes over regulatory standards. The United States government takes the final product as the regulatory object and follows the “principle of case analysis” for the supervision of CRISPR-Cas9 editing crop products, which is cooperatively regulated by the United States Department of Agriculture, the Environmental Protection Agency and the Food and Drug Administration. At present, a variety of CRISPR-Cas9 editing crops have passed the review of the three departments, exempting from the regulation of GMO (Pan et al., 2021).

EU countries proposed to take process as the supervision object for CRISPR-Cas9 products. As long as transgenesis is involved in the development process, they should be strictly regulated regardless of the existence of the exogenous components in the final plants. In 2018, the European Court of Justice ruled that Gene-edited crops should follow GMO regulatory procedures (Wang et al., 2020b).

Regulation three of the Regulations on the Safety Management of Agricultural Gmos, promulgated in 2001 and revised in 2017, stipulates that “agricultural genetically modified organisms as mentioned in this regulation refer to the animals, plants, microorganisms, and their products used in agricultural production or agricultural products processing, which has been changed in the composition of the genome by using the genetic engineering technology.” Gene editing technology is one of the genetic engineering technologies. Therefore, it seems that gene editing crops should be supervised like the genetically modified organisms, but there is no explicit stipulation on how to supervise gene editing crops. Moreover, some Chinese scholars believe that the regulation of CRISPR-Cas9 editing crops without exogenous DNA such as SDN-1 according to traditional genetically modified organisms is likely to hinder the development of this technology (Fan et al., 2020). In recent years, Chinese scientists Gao Caixia, Academician Cao Xiaofeng, Academician Liu Yaoguang, Academician Zhu Health, and Li Jiayang have put forward reasonable suggestions on the supervision of gene editing crops through the media or papers (Wang et al., 2020b).

## The Prospect of CRISPR-Cas9

Since the discovery of regularly spaced repeats in 1978, the mechanism of action of the CRISPPR/Cas9 system has been elaborated in 2012, and CRISPPR/Cas9 has gradually penetrated into the field of biology. As a sequence-specific nuclease, after continuous development and improvement by scientists, it basically surpasses ZFNs and TALENs in terms of application efficiency and extensiveness. The main advantages of the CRISPPR-Cas9 system are its strong specificity, high efficiency, simple design, low cost, and the ability to edit multiple genes simultaneously. It is precisely because of the powerful advantages of CRISPPR-Cas9 that the system has attracted widespread attention from biological researchers around the world as soon as it came out. Therefore, in the 10 years from 2012 to 2022, the CRISPPR-Cas9 system has been rapidly developed and expanded. In addition to the traditional CRISPR-Cas9 system based on DNA double-strand breaks, there are base editors and prime editors based on single-strand breaks, as well as CRISPR-dCas9 systems that do not cause DNA breaks.

**Figure 1:**
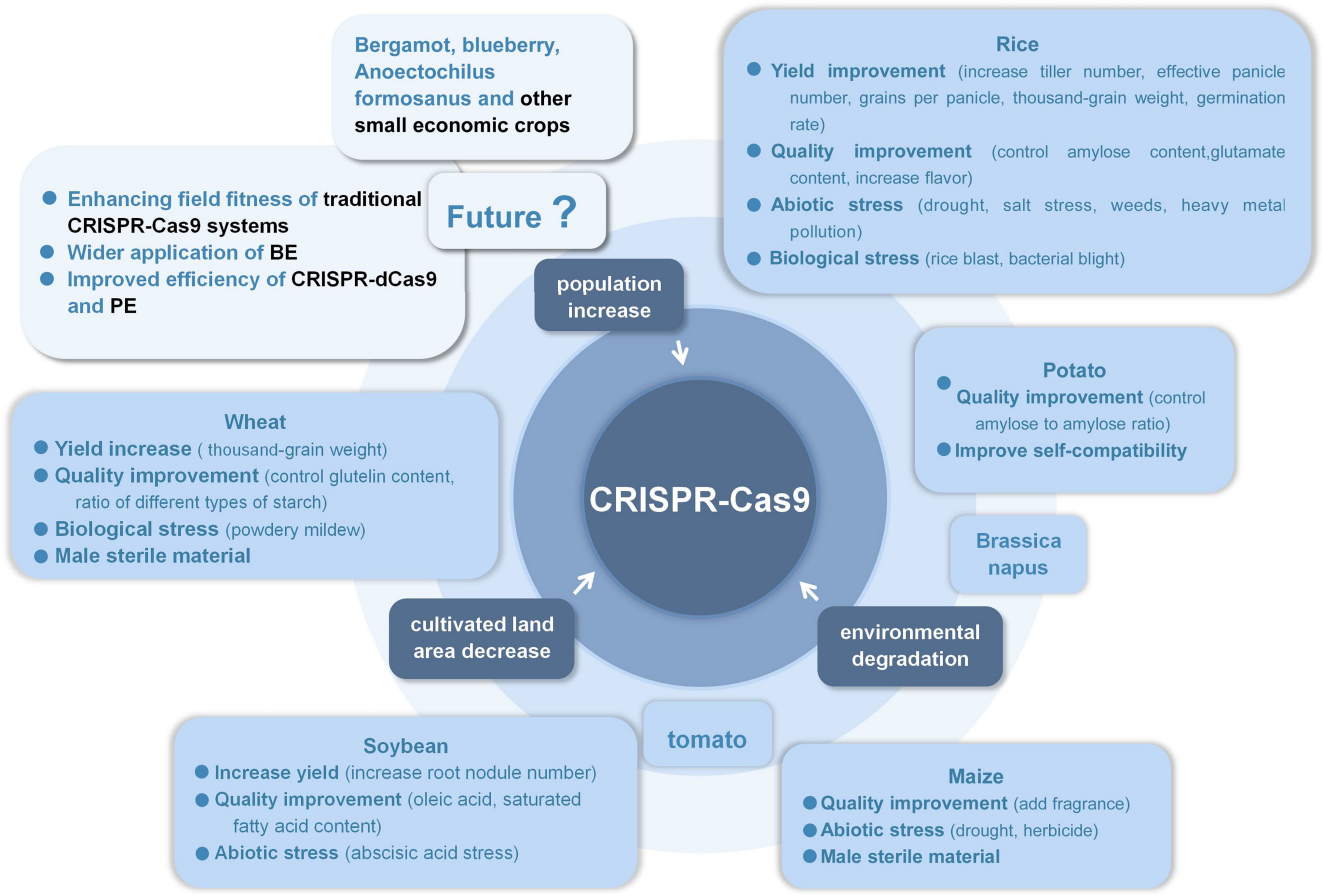
The prospects of CRISPR-Cas9 in crop improvement.

Traditional CRISPPR-Cas9 system has been successfully used in tens of crop improvement: (1) model plant *Arabidopsis thaliana* and tobacco, (2) food crops like rice, wheat, maize, sorghum, potato, and cassava, (3) oil crops like soybean, peanut, rapeseed, and sesame, (4) vegetable crops like tomato, cabbage, carrot, mushroom, and luffa, (5) fruit crops like apple, banana, pear, grape, strawberry, citrus, sweet orange, and litchi, (6) tree crops like cotton, poplar, larch, tea, rubber tree, coffee, and cocoa, and (7) flowers and herbs like petunia, chrysanthemum, lily, phalaenopsis, torenia fournieri, hemerocallis, liverwort, alfalfa, and panax, which reflects the pretty wide applicability of this technique (Liu et al., 2017, 2021c; Yao et al., 2017; Shi et al., 2018; Chen, 2020; Huang et al., 2020; Li and Xiao, 2020; Liu, 2020; Lv et al., 2020; Situ et al., 2020; Cao, 2021; Chen et al., 2021; Huang, 2021; Kan et al., 2021; Li, 2021b; Liao et al., 2021; Wang et al., 2021; Yang, 2021; Zhou et al., 2021a). Figure 1 summarizes the past applications of CRISPR-Cas9 technology and prospects its potential applications in the future.

Especially in the application of important crops such as rice, wheat, corn, soybean, and potato, it has been widely used in increasing yield, improving crop quality, obtaining biological resistance and abiotic resistance, and obtaining male sterile materials, etc. The traditional double-strand break-dependent CRISPR-Cas9 system significantly improved most traits. On the one hand, the application of these excellent varieties created in crop breeding should be strengthened to make them suitable for field planting and effectively reduce the burden on farmers. On the other hand, applying the huge application potential of CRISPR-Cas9 technology to more crops, such as bergamot, clematis, blueberry, and other small commercial crops, first, it can increase the yield and meet the greater market demand. Second, it can improve the taste and nutritional value to meet the quality requirements of human beings. In addition, it can improve the abiotic resistance of crops and cultivate different varieties for the same crop to adapt to various climatic conditions, so as to reduce transportation costs and reduce the price of rare crops.

As a tool for accurate gene regulation, the CRISPR-dCas9 system has great potential for development, and has already been used in *Arabidopsis*, rice, and tobacco. However, there is still a problem of low efficiency, which needs to be further optimized by researchers.

As a precise base-changing system, BE have created varieties with improved traits in crops such as rice, wheat, *Arabidopsis*, and rapeseed. The application of herbicide resistance is particularly prominent. *ALS* genes in rice, wheat, tomato, potato, and watermelon were edited to make crops tolerant to IMI.

As PE can perform any kind of substitution to the four bases, it has great application potential. At present, guided editing has been completed in rice and wheat, but faced with the challenge of low editing efficiency. At present, researchers are constantly optimizing the guided editing system in order to achieve higher editing efficiency in the future.

Although CRISPR-Cas9 technology faces challenges such as introducing foreign genes and causing off-target effects, these challenges have been gradually overcome through improvements by researchers. In view of the introduction of foreign genes by CRISPR-Cas9 technology, the study found that the foreign genes can be lost through progeny separation, so as to obtain non-transgenic offspring, or by assembling sgRNA and Cas9 into a ribonucleoprotein complex, the source of foreign genes can be avoided. For off-target effects caused by CRISPR-Cas9 technology, it could be reduced by optimizing sgRNA and modifying Cas protein. It is believed that more and more efficient optimization measures will be applied to crop improvement in the near future.

## Author Contributions

YR and KW are the content designers. XY wrote the manuscript. YR and CW revised the manuscript. CP looking up references. All authors contributed to the article and approved the submitted version.

## Funding

This work was supported by the National Natural Science Foundation of China (U20A2030), the State Key Laboratory of Rice Biology, China (20200102), and Agricultural Science and Technology Innovation Program of CAAS, Key Laboratory of Rice Genetics and Breeding, Guangxi (2018-15-Z06-KF12).

## Conflict of Interest

The authors declare that the research was conducted in the absence of any commercial or financial relationships that could be construed as a potential conflict of interest.

## Publisher’s Note

All claims expressed in this article are solely those of the authors and do not necessarily represent those of their affiliated organizations, or those of the publisher, the editors and the reviewers. Any product that may be evaluated in this article, or claim that may be made by its manufacturer, is not guaranteed or endorsed by the publisher.
